# Epilepsy in children: quality of life and disease control

**DOI:** 10.3389/fneur.2025.1692379

**Published:** 2025-12-05

**Authors:** Miral A. Almomani, Basima A. Almomani, Roaa B. Banikhaled, Roa'a N. Elayyan, Yumna H. Abu Abbas, Hanan Al Thiabat, Hashim Al-Qudah

**Affiliations:** 1 Department of Pediatrics and Neonatology, Faculty of Medicine, King Abdullah University Hospital, Jordan University of Science and Technology, Irbid, Jordan; 2 Department of Clinical Pharmacy, Faculty of Pharmacy, Jordan University of Science and Technology, Irbid, Jordan; 3 Faculty of Medicine, Jordan University of Science and Technology, Irbid, Jordan

**Keywords:** children, disease control, epilepsy, quality of life, comorbidities, antiepileptic medication

## Abstract

**Introduction:**

Childhood epilepsy negatively affects health, quality of life (QoL), and character development.

**Methods:**

A cross-sectional online survey was conducted among pediatric epilepsy patients in Jordan. Candidates were identified through a hospital database. The shortened Quality of Life in Childhood Epilepsy Questionnaire (QOLCE-55) assessed QoL. Disease control was defined as seizure-free for 1 year. Clinical characteristics, QoL, disease control, and their determinants among pediatric epilepsy patients in Jordan were assessed.

**Results:**

A total of 178 patients participated. The mean health-related QoL (HRQoL) score was 50.74 ± 22.54, highest in emotional wellbeing (60.75 ± 19.40) and lowest in physical functioning (44.75 ± 24.06). Higher HRQoL was significantly associated with older age at diagnosis (β = 1.668, *P* = 0.001), absence of comorbidities (β = −10.851, *P* = 0.006), fewer seizures annually (β = 7.572, *P* = 0.001), and use of fewer antiepileptic medications (β = −10.665, *P* = 0.002). Only 42.1% had controlled epilepsy. Disease control was associated with older age (OR = 1.134, 95% CI: 1.011–1.272, *P* = 0.032) and higher QoL (OR = 1.035, 95% CI: 1.014–1.057, *P* = 0.001). Uncontrolled seizures were linked to caregiver exhaustion (OR = 0.34, 95% CI: 0.155–0.743, *P* = 0.007) and polytherapy (OR = 0.397, 95% CI: 0.179–0.882, *P* = 0.023).

**Conclusion:**

Epilepsy significantly reduces QoL in children. Better QoL and seizure control were linked to older age, absence of comorbidities, fewer seizures, and reduced medication use. Caregiver support and minimizing polytherapy may enhance both QoL and disease control.

## 1 Introduction

Epilepsy is defined as a chronic brain disorder marked by an enduring proclivity for recurrent epileptic seizures as well as the neurobiological, cognitive, psychological, and social repercussions of this disorder (1, 2). At this given time, Epilepsy affects ~50 million people worldwide (3). In developed countries, it is estimated that there are 3.4–11.3 cases per 1,000 children (3, 4). In Arab countries, the median lifetime prevalence of epilepsy in Arab countries was found to be 6.9 per 1,000 (5). Regarding Pediatrics, epilepsy was found to have evident detrimental impacts on a child's health and quality of life (2). Therefore, this can have a negative impact on the formation of a healthy character during childhood, which is influenced directly by the development of successful peer relationships and appropriate degrees of independence (2). As a result, both parents and their affected children may find it difficult to cope with this disease (2).

Epilepsy affects all elements of quality of life in children and adolescents, as well as their families, to varying degrees (6). Several studies were carried out to observe the different factors that significantly affect pediatrics QoL among different countries (6). Different factors across different studies were reported to be associated with QoL such as gender of patient, duration of epilepsy, the type, frequency, and intensity of seizures, comorbidities, the number and side effects of anti–seizure drugs, duration of treatment, the existence of comorbidities, parental anxiety, and family socioeconomic level all influence the quality of life of people with epilepsy (2, 3, 7–9).

Numerous studies conducted globally have examined the quality of life in children with epilepsy and the significant negative impact the condition can have on both the patients and their families (1–3, 10–14). These studies consistently highlight the physical, emotional, and social challenges faced by children with epilepsy and the burden placed on their caregivers. In contrast, research focusing on the quality of life in pediatric epilepsy patients within Arab countries remains limited. The majority of existing studies have been conducted in Saudi Arabia, including those by (8, 15, 16), leaving a gap in data from other countries in the region.

In Jordan specifically, studies on pediatric epilepsy have largely focused on clinical and epidemiological aspects. For instance, Al-Qudah et al. (17) explored the types and etiology of epilepsy, and clinical factors influence both quality of life and disease control while Masri et al. (18) assessed parental knowledge and attitudes. More recently, Al-Ghawanmeh (19) reported that seizure control was achieved in 73% of pediatric patients and identified cultural factors—such as family size and family history—as contributors to increased caregiver burden. However, none of these studies have directly examined the quality of life in pediatric epilepsy patients or how various sociodemographic. Given this significant gap, the present study aims to assess patient-reported outcomes related to quality of life and seizure control in children with epilepsy in Jordan. By evaluating the impact of sociodemographic and clinical variables on these outcomes, this study seeks to provide critical insights that can guide the development of targeted, evidence-based interventions. Ultimately, these findings can support improved care strategies and better quality of life for pediatric epilepsy patients and their families in Jordan.

## 2 Method

### 2.1 Study design

A cross-sectional survey was conducted in Jordan on pediatric patients with epilepsy via online questionnaires from June to August 2024. Mothers of children with epilepsy attending King Abdullah University Hospital (KAUH) were invited to participate in the survey. Inclusion criteria included pediatric patients aged from 4 to 18 years with confirmed diagnosis of epilepsy by neurologist based on International League Against Epilepsy (ILAE) definition (20). Participants who were newly diagnosed with epilepsy, had recently discontinued their antiepileptic drug (AED) regimen, or had passed away were excluded from the study. The ethical approval from institutional review board (IRB; Ref number 2023/279) was obtained. A list of patients who visited KAUH/pediatrics Neurology Department between January 2021 and June 2024 was collected from computerized hospital databases (*n* = 849). Those who met the inclusion criteria were contacted via WhatsApp. Informed consent was obtained from the parents at the beginning of the questionnaire. Ten minutes was the expected time to complete the questionnaire.

### 2.2 Outcome measures

The shortened Quality of Life in Childhood Epilepsy Questionnaire (QOLCE-55) was developed and validated by Goodwin et al. (21). It is a parent-reported instrument specifically developed to assess the health-related quality of life (HRQoL) of children with epilepsy, aged 4–18 years (21). The Arabic version of this measure was validated by Khalil et al. (22) and was used in the current study. The questionnaire consists of four domains of life: cognitive (22 items), emotional (17 Items), social (seven items), and physical (nine items). In our study, the internal consistency of the questionnaire measured using Cronbach's alpha was 0.97. Responses are rated on a six-point Likert scale, ranging from 0 (very often) to 5 (not applicable). Each item's score was converted linearly into a 0–100 value, such as 0 = 0, 1 = 25, 2 = 50, 3 = 75, and 4 = 100. The scores were then summed up, and an average was determined, with higher scores reflecting better HRQoL.

Disease control was assessed based on disease- free for a year. Chen et al. (23) defined seizure control as achieving seizure freedom over study period of 1 year. The following demographic and clinical data were collected from mothers of patients age, gender, family monthly income, residency, comorbid condition, education of parents, employment, health insurance, age of diagnosis, chronic disease, type of epilepsy, duration of seizure, disease intensity, frequency of seizure, number of medications, side effects, and seizure control.

### 2.3 Statistical analysis

All data gathered from participants was coded and then imported into IBM SPSS Statistics software version 22 (Armonk, NY: IBM Corporation). Based on the data acquired, various statistical analyses were applied as appropriate. In which, the independent *t*-test, analysis of variance (ANOVA) and Pearson correlation were used to assess factors associated with HRQoL. While factors associated with epilepsy control were examined using the independent *t*-test, Chi-square test and Mann Whitney test. Normality of data was assumed if the histogram showed a symmetrical distribution, the Quantile-Quantile plot showed points with no deviation from reference line and the Kolmogorov-Smirnov test and Shapiro-Wilk test yielded a *P*-value >0.05. Levene's test was also used to analyze variance, with a *P*-value of >0.05 indicating that the variance was the same in all groups. All variables with *P*-value < 0.1 in the univariate analysis were included in the multivariate analysis (linear or binary logistic regression). As, linear regression was used to investigate factors associated with HRQoL while epilepsy control [yearly (controlled) vs. daily/weekly/monthly (uncontrolled)] associated factors were examined using binary logistic regression. A histogram of the residuals was employed to test the normality of regression, with a normal distribution assumed if the plot is symmetrical. Homoscedasticity and linearity were investigated using a scatterplot of residuals vs. predicted values. When the points are symmetrically distributed around the horizontal line and have a constant variance across the plot, this indicates linear and homoscedastic data. To evaluate other assumptions, we employed the Variance Inflation Factor (VIF) to measure multicolinearity, with values < 5 indicating no multicolinearity, and the Durbin Watson value to examine data independence, with values ranging from 1 to 3 meaning an independent residual. During the analysis, the presence of influential or outlier points was also determined using Cook's distance value; a value smaller than one indicates that there are no significant outliers or influential points. *P*-value < 0.05 was considered as statistically significant.

## 3 Results

### 3.1 Baseline characteristics

**Figure 1:**
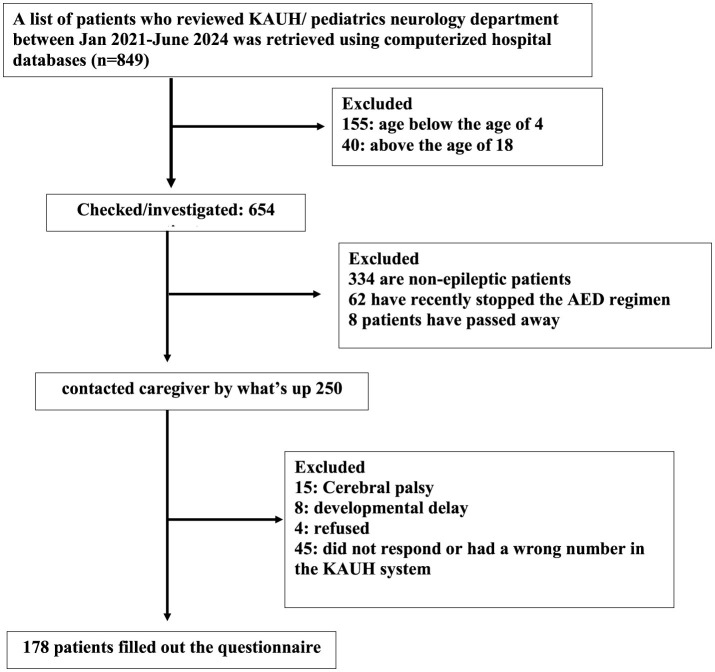
Flow chart for study recruitment.

A total of 159 patients were removed due to age range (< 4 years to >18 years). Of the remaining 654 individuals on the list, 404 were removed because they were not epileptic (*n* = 334), had recently quit using AED (*n* = 62), or had died (*n* = 8). A total of 250 epileptic patients satisfied the study's inclusion criteria. Of these, 72 were eliminated for various reasons, leaving 178 patients to be recruited and included in the final analysis. Figure 1 presents detailed data.

Patients had a mean age of 10.73 ± 4.06 years with more than half of the patients were males (60.7%) and insured (73%). Half of the patients' parents lived in the city (50%) and had a monthly income of < 600 JD (50.6%). More than half of them (64%) found caring for epileptic patients to be exhausting, and 46.6 % had only completed school education. The median age of diagnosis was 6 years. A third of the patients have a focal onset seizure (37.6%), most of the patients use only one medication (57.9%), and the majority do not have another chronic disease (80.3%). Around one quarter of patients had seizure episodes occurred on yearly basis (42.1%), with 50.3% having mild episodes, and the majority of patients' seizures lasted < 5 min (70.7%). Less than half of patients (41.6%) suffered from medication side effects. Detailed data is presented in Table 1.

**Table 1:** Demographic and clinical characteristics.

**Demographic characteristics^a^**	***N* = 178 (%)**
Age (years)^b^	10.73 ± 4.06
**Gender**	
Female	70 (39.3%)
Male	108 (60.7)
**Relationship to the patient**	
Father	72 (40.4)
Mother	101 (56.7)
Other relatives (grandparents, uncles, aunts)	5 (2.8)
**Family monthly income**	
< 600 JD	90 (50.6)
≥600 JD	88 (49.4)
**Residency**	
City	89 (50)
Village	89 (50)
**Parents' education**	
School education	83 (46.6)
University education	95 (53.4)
**Insurance**	
No	48 (27)
Yes	130 (73)
**Caring for patient is exhausting job for caregiver**	
No	64 (36)
Yes	114 (64)
**Clinical characteristics**	
Age at diagnosis (years)^c^	6 [2–9]
**Type of epilepsy**	
Focal onset	67 (37.6)
Generalized onset	51 (28.7)
Unknown onset	60 (33.7)
**Duration of seizure**	
< 5 min	123 (70.7)
≥5 min	51 (29.3)
**Number of antiepileptic medications**	
One medication	103 (57.9)
≥ two medications	75 (42.1)
**Side effects of medications**	
No	104 (58.4)
Yes	74 (41.6)
**Seizure intensity**	
Mild	80 (50.3)
Moderate	48 (30.2)
Severe	31 (19.5)
**Number of seizure occurrence**	
Daily	43 (24.2)
Weekly + monthly	60 (33.7)
Yearly	75 (42.1)
**Comorbid conditions**	
No	143 (80.3)
Yes	35 (19.7)

^a^All data was expressed as n (%) of participants unless otherwise indicated.

^b^Data were described as mean ±SD—(normal distribution).

^c^Data were described as median [interquartile range]—(skewed distribution).

### 3.2 Quality of life

As presented in Table 2, the total QoL mean among patients was 50.74 ± 22.54 out of 100, with the greatest score was on the emotional scale at 60.75 ± 19.40 and the lowest was on the physical scale at 44.75 ± 24.06. Multivariate analysis (Table 3) revealed that older children at diagnosis (β = 1.668, *P*-value = 0.001), lack of comorbid conditions (β = −10.851, *P*-value 0.006), annual occurrence of seizure (β = 7.572, *P*-value = 0.001), and fewer number of epilepsy medications (β= −10.6651, *P*-value 0.002) were associated with better HRQoL and wellbeing.

**Table 2:** Mean score for all scales.

**Scale**	**Number of questions**	**Mean ±SD**	**Median (IQR)**
Cognitive functioning^a^	22	48.13 ± 30.32	51.14 (21.59–71.31)
Emotional functioning^a^	17	60.75 ± 19.40	63.2353 (49.26–73.53)
Social functioning^b^	7	60.05 ± 28.50	60.71 (42.86–85.71)
Physical functioning^a^	9	44.75 ± 24.06	44.44 (27.78–63.19)
Total score^a^	55	50.74 ± 22.54	52.98 (35.90–66.33)

^a^Data was normally distributed.

^b^Data had a skewed distribution.

**Table 3:** Multivariate analysis for factors associated with HRQoL.

**Baseline characteristics**	***B***	***P*-value**
Age	−0.306	0.544
Age at diagnosis	1.668	**0.001**
Monthly income	0.375	0.904
**Comorbid conditions**		
No (reference)	−10.851	**0.006**
Yes		
**Seizure occurrence times**		
Daily (reference)	7.572	**0.001**
Weekly+ monthly		
Yearly		
**Seizure intensity**		
Mild (reference)	−0.836	0.675
Moderate		
Severe		
Number of antiepileptic medications	−10.665	**0.002**
**Exhausting task**		
No (reference)	0.976	0.776
Yes		

Bold values indicate statistically significant associations (*P*-value < 0.05) in the multivariate analysis.

### 3.3 Disease control

Less than half of patients (42.1%) were found to have controlled disease. As presented in Table 4, controlled epilepsy was found to be associated with older age (OR = 1.134, 95% CI = 1.011–1.272, *P*-value = 0.032) and having a higher quality of life (OR = 1.035, 95% CI = 1.014–1.057, *P*-value = 0.001). On the other hand, uncontrolled seizures were associated with parents who reported that caring for their children's seizures was an exhausting task (OR = 0.34, 95% CI = 0.155–0.743, *P* = 0.007), as well as with the need for higher number of antiepileptic drugs (OR = 0.397, 95% CI = 0.179–0.882, *P* = 0.023).

**Table 4:** Multivariate analysis of factors associated with controlled epilepsy.

**Characteristics**	**OR**	**95% CI**	***P*-value**
Age of patient	1.134	1.011–1.272	**0.032**
Age at diagnosis	0.919	0.819–1.032	0.155
**Duration of seizure**			
< 5 min (reference)	2.031	0.922–4.474	0.079
≥5 min			
**Exhausting task**			
No (reference)	0.34	0.155–0.743	**0.007**
Yes			
**Number of antiepileptic medications**			
One medication (reference)	0.397	0.179–0.882	**0.023**
Two or more medication			
QoL score	1.035	1.014–1.057	**0.001**

Bold values indicate statistically significant associations (*P*-value < 0.05) in the multivariate analysis.

## 4 Discussion

This study is the first to assess quality of life and disease control in children with epilepsy in Jordan. Key findings include: (i) patients exhibited a moderate quality of life, (ii) fewer than half achieved seizure control, and (iii) multiple factors influenced both QoL and disease control. The current study showed that seizure onset in patients averaged 5 years, similar to Hussain et al. though diagnosis age was higher than in Elsakka et al., likely due to population differences (12, 24). Most seizures lasted under 5 min, consistent with previous findings (25, 26). One-third had focal onset epilepsy, while other studies reported more generalized types, possibly due to younger subjects (27, 28). Unlike Wagner et al. (29) most of our cases were mild, possibly due to their smaller sample. Annual seizures were more common here, contrasting with studies involving cognitively impaired patients or those on long-term medication (8, 30). Most patients had no comorbidities, consistent with prior findings (31, 32). Over half used monotherapy, differing from some but matching others, possibly due to COVID-19–related factors (28, 31).

Our study found that the physical domain of QoL which assessed the impact of epilepsy on various physical activities both indoors and outdoors, was the most affected domain. This finding aligns with studies (14, 33) but contrasts with another study (13) where the cognitive function domain has the least QOL mean score and was the most impaired domain. Furthermore, the lack of nearby swimming facilities, particularly for individuals living outside the city, may further contribute to this limitation. Our study also showed that the least comprised domains are the emotional and social domains. Similarly, Marghalani et al. (16) results show that the highest QOL mean scores were also in both emotional and social domains.

### 4.1 Factors influencing quality of life

Our findings show that older age at diagnosis is significantly associated with lower quality of life (QoL) consistent with a prior study (9). In contrast, Pachange et al. (33) found that earlier seizure onset was linked to poorer overall QoL and negative impacts on the social domain. This may be explained by how differences in “age at diagnosis” and “age of seizure onset” shape the patient's experience—late diagnosis delays treatment, while early onset disrupts development—both ultimately lowering QoL through distinct mechanisms. In addition, the poorer QoL observed in younger participants may be attributable to their higher likelihood of experiencing more severe clinical conditions in this age range, such as epileptic encephalopathy (EE) and developmental and epileptic encephalopathy (DEE). In the current study, frequency of seizure was found to be significantly associated with a lower Qol consistent with Okazaki et al. (34) implying the need for better seizure control to achieve improved Qol as noted in the previous study (26). Children with poorly controlled seizures, along with their caregivers, often face increased stress, anxiety, depression, sleep difficulties, and social stigma compared to those managing seizures more effectively. Poor adherence to treatment can worsen seizure control, leading to a further decline in quality of life. Our study found that comorbidities significantly reduced quality of life, consistent with findings from previous research (9, 35). This could possibly be due to the increased physical and cognitive burden that comorbid children often face, multiple emotional and behavioral challenges and being considered a healthcare burden due to frequent hospital visits and healthcare utilization. In this study, patients on multiple antiepileptic drugs were found to have a lower QOL which coincides with Nagesh et al. (13) where patients with poly therapy had poor health perceptions, limited social interaction, lower energy levels due to seizure worry and health discouragement.

### 4.2 Factors influencing disease control

Most of our patients have uncontrolled diseases which are consistent with Adal et al. but not with Poudel et al. (36, 37). The possible rationale for this could be Poudel et al.'s distinct definition of disease control, which defines disease control as the absence of seizures for 2 months, which is less than the time frame indicated in our study, which is disease- free for a year or more. The binary logistic regression revealed factors associated with disease control. Patients who are older in age have a significantly better controlled epilepsy which contradict Adal et al. (36) findings. This could be explained that older patients have higher levels of maturity, independence, comprehension, awareness of importance of therapy thus better disease control. Riechmann et al. found that parents of epileptic patients are negatively impacted by disease as they have a poorer QoL, higher disease burden, and anxiety (26, 38). In our study, most parents reported that caring for their children was an exhausting and stressful task, and this was associated with poorer epilepsy control. Furthermore, individuals receiving monotherapy have better epilepsy control than those receiving polytherapy, in line with Adal et al. (36). The use of polytherapy may lead to poor adherence and thus worsened disease control (36). Additionally, in our study, patients appear to have better quality of life when they have a controlled disease (less frequent seizure) which is consistent with previous studies (34, 38).

### 4.3 Limitation

The current study has some limitations. First, because our study was cross sectional, no causal relationship could be made. Second, our study was based on self-report questionnaire which has the probability of recalling bias. Third, the study was conducted at a single medical center in Irbid, Jordan, which could impact the generalizability of the results collected. Using a parent-reported questionnaire instead of a patient-reported one may have introduced bias. Parent perception of their children's health and wellbeing may differ from the children's own perspective.

## 5 Conclusion

This is the first study to examine both quality of life and disease control, along with their predictors, in children with epilepsy in Jordan. Our findings reveal a compromised QoL among this population, emphasizing the need for treatment plans that include regular evaluations of the child's overall wellbeing. The strong association between reduced caregiver burden and improved QoL and seizure outcomes suggests the importance of incorporating caregiver support into epilepsy management. Future studies should evaluate the effectiveness of such interventions. Additionally, the observed link between fewer medications and better outcomes underscores the need for routine review of treatment regimens, with consideration for minimizing polytherapy when appropriate. Finally, involving patients and families in treatment decisions may enhance adherence, reduce caregiver stress, and lead to improved clinical outcomes.

## Data Availability

The raw data supporting the conclusions of this article will be made available by the authors, without undue reservation.
